# Exosomes and cancer - Diagnostic and prognostic biomarkers and therapeutic vehicle

**DOI:** 10.1038/s41389-022-00431-5

**Published:** 2022-09-15

**Authors:** Xia Wang, Lu Tian, Jingyi Lu, Irene Oi-Lin Ng

**Affiliations:** 1 Department of Pathology, The University of Hong Kong, Hong Kong Special Administrative Region, China; 2 State Key Laboratory of Liver Research, The University of Hong Kong, Hong Kong Special Administrative Region, China

**Keywords:** Cancer, Tumour biomarkers

## Abstract

Exosomes belong to a subpopulation of extracellular vesicles secreted by the dynamic multistep endocytosis process and carry diverse functional molecular cargoes, including proteins, lipids, nucleic acids (DNA, messenger and noncoding RNA), and metabolites to promote intercellular communication. Proteins and noncoding RNA are among the most abundant contents in exosomes; they have biological functions and are selectively packaged into exosomes. Exosomes derived from tumor, stromal and immune cells contribute to the multiple stages of cancer progression as well as resistance to therapy. In this review, we will discuss the biogenesis of exosomes and their roles in cancer development. Since specific contents within exosomes originate from their cells of origin, this property allows exosomes to function as valuable biomarkers. We will also discuss the potential use of exosomes as diagnostic and prognostic biomarkers or predictors for different therapeutic strategies for multiple cancers. Furthermore, the applications of exosomes as direct therapeutic targets or engineered vehicles for drugs are an important field of exosome study. Better understanding of exosome biology may pave the way to promising exosome-based clinical applications.

## Introduction

Extracellular vesicles (EVs) are important mediators in intercellular communication, both local and systemic, by transferring their components among different cells. EVs are heterogeneous membrane-bound vesicles that are classified by size, density, and cellular origin. Based on their biogenesis, EVs consist mainly of three sub-classes including endosome-origin exosomes, plasma membrane-derived microvesicles (‘ectosomes’), and apoptotic bodies. Exosomes are 30–200 nm in size while microvesicles are larger, ranging from 50 nm to 1000 nm. Unlike microvesicles, which are formed by direct budding of the plasma membrane, exosomes are formed from the invagination of the endosome membrane and released via fusion of the multivesicle bodies (MVB) and the plasma membrane. EVs synthesized by some particular cell types (such as T cells) may utilize different pathways, but the aforementioned nomenclature is most commonly applied [1]. Compared to microvesicles, the biosynthesis of exosomes is a highly specific and regulated process.

As exosomes are released by cells, they inherit the cellular components to exert the roles of their parental cells. The exchange of biological materials and signals influence the multifaceted functions of tumor cells, such as stemness properties, proliferation, distant metastasis, drug resistance, and immune response [2–5]. In the tumor microenvironment (TME), the stromal cells can generate exosomes that modulate the malignant behavior and response to stress conditions of the tumor cell [6]. Evidence has shown that tumor cells can foster an immunosuppressive environment in situ or prime a favorable pre-metastatic niche in distant organs through exosome release [7, 8]. On the other hand, exosomes derived from immune cells play more complicated roles due to their polarization properties. Several lines of evidence showed that exosomes from cytotoxic lymphocytes and antigen-presenting cells (APCs) could enhance immune response [9, 10].

As nano-sized particles, exosomes are secreted into the circulation. As such, many types of the exosomal components could be used for diagnosis, prognosis, and disease monitoring. Not only the quantity of the exosomes but also their compositions (proteins, miRNA, and lncRNA) are important in cancer diagnosis [11, 12]. Exosomal markers (CD63, CD81, CD9) [13, 14], some tumor antigens (CEA, CA125) [15, 16] and stemness markers (EpCAM, CD24) [17, 18] are elevated in circulating exosomes and may have diagnostic values. For instance, clinically, exosomal PD-L1 has been shown to be a potential predictor of response for anti-PD-1 therapy in patients with melanoma and non-small cell lung cancer (NSCLC) [19, 20].

The membrane composition and low immunogenicity make exosomes a promising way for drug delivery. Some exosomes derived from immune cells could facilitate tumor cell elimination [21]. Moreover, chemotherapy drugs or siRNAs targeting oncogenic signaling could be loaded in exosomes to exert their anti-tumor roles [22]. Studies have also revealed that genetically engineered parental cells can release exosomes with corresponding changes to impair tumor cell growth and sensitize tumor cells to drugs [23].

In this review, we will summarize the updated findings and progresses of exosome-related research in the cancer field. Among the many aspects of exosomes in cancers, we will highlight the main mechanisms of exosome biosynthesis, signify the interlocking and bidirectional effects of exosomes in the TME, and address their diagnostic and prognostic values as well as therapeutic potentials.

## Exosome biogenesis and uptake pathways

**Fig. 1:**
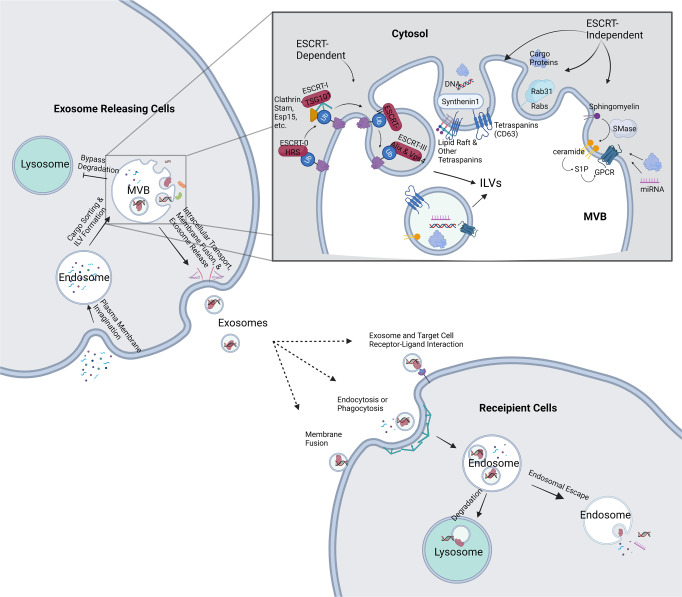
Overview of exosome biosynthesis and uptake pathways. Intraluminal vesicles (ILVs) are synthesized from endosomes through cargo-sorting machineries and concurrent membrane invagination. The ILVs-containing multi-vesicle bodies (MVBs) then bypass lysosomal degradation and are transported to the plasma membrane, where they undergo vesicle docking, resulting in the release of exosomes into the extracellular space. Exosomes interact with the recipient cells by receptor-ligand interaction, membrane fusion, or phagocytosis or endocytosis. Being phagocytosed or endocytosed, exosomes bypass endosome-lysosome pathway and release their contents in the cytoplasm. Four exosomal protein sorting machineries (ESCRT-, tetraspanins-, Rabs-, and ceramide- dependent machineries) are depicted in the zoom-in view.

As part of the endocytic pathway, exosome biosynthesis begins with cargo recruitment, membrane invagination of MVB, and formation of intraluminal vesicles (ILVs) within the lumen of MVB. Upon maturation of the ILVs, exosomes are released into the extracellular space after MVBs have fused with the plasma membrane (Fig. 1).

### Exosomal cargo sorting and intraluminal vesicles (ILV) formation

Exosomal sorting machineries recruit cargos as the ILVs are formed. So far, four sorting machineries have been proposed. The well-established or canonical sorting machinery involves the endosomal sorting complexes required for transport (ESCRT) machinery, which comprises four ESCRT complexes (ESCRT-0, -I, -II, and -III). This pathway starts with Hrs (hepatocyte growth factor-regulated tyrosine kinase substrate) protein (within ESCRT-0), recognizing ubiquitinated proteins. Hrs, with the aid of associated proteins (such as Stam, Esp15, and clathrin), sequesters cargo proteins and recruits Tsg101 (within ESCRT-I), and then recruits ESCRT-II. Along with other accessory proteins, ESCRT-III is recruited; the whole complex drives the membrane budding as well as vesicle scission [1, 24].

Since ESCRT-depleted cells are still able to generate ILVs, other ESCRT-independent ILV formation pathways must also exist. So far, three ESCRT-independent machineries have been hypothesized and require ceramide, tetraspanins (CD63), and Rabs (Rab31), respectively. In ESCRT-deficient cells, sphingomyelinase hydrolyzes sphingomyelin into ceramide. Ceramide induces the formation of raft-based microdomains, thus promoting membrane curvature and subsequent budding [25]. In addition, ceramide can also be further metabolized into sphingosine 1-phosphate, which is coupled to Gi protein and can activate cargo (CD63) sorting and exosome maturation [26].

### Intracellular transport, release, and uptake of exosomes

After the sorting processes get completed, the MVBs bypass lysosomal fusion through an active mechanism. The bypass mechanism is not clear, but there is hypothesis that the exosomal contents (such as Rab7) could determine the fate of MVBs [27–29]. Then the MVBs are transported along the cytoskeleton to the plasma membrane by molecular motors consisting of kinesins, dynein and their adaptor GTPases [1]. The final step of exosome biosynthesis involves the fusion of MVB with the plasma membrane and the release of exosomes into the extracellular space. This process is accomplished by Rab GTPases, v-SNAREs (such as vesicle-associated membrane protein 7 and synaptobrevin homolog YKT6) [30], tethering proteins (such as early endosome antigen 1), and synaptotagmins [31]. The targeted delivery to the designated cells is accomplished by different compositions of the exosomal envelope [32], such as integrin proteins [33]. Exosomes can interact with the recipient cells via various mechanisms such as binding of exosomal surface proteins to receptors on the recipient cell membrane, fusion with the cellular plasma membrane, and being endocytosed or phagocytosed. If endocytosed and phagocytosed, the internalized exosomes will result in endosomes. Exosomes will once again escape the endosomal/lysosomal pathway, leading to exosomal cargo release and their functions in the cytosol [34, 35].

## Exosomes isolation and detection

### Exosomes isolation

Exosomes can be extracted from multiple sources, including cell culture medium, body fluids and tumor tissues. Methods to harvest exosomes include differential ultracentrifugation (UC), density gradient fractionation, polymeric precipitation, ultrafiltration, size-exclusion chromatography (SEC), and immunoaffinity isolation [36]. The cell culture medium is the most widely used material, and UC is the most commonly used isolation method. UC usually yields highly purified exosomes. Polymeric precipitation produces the highest yields but also the heaviest contamination. Other methods like ultrafiltration and SEC can trap or separate particles by physical barriers. In addition, immunoaffinity isolation based on the characteristic surface proteins are used to study specific subgroups of exosomes. Antibodies conjugated with beads can select the desired exosomes (immuno-enrichment) or trap unwanted exosomes (immune-depletion). This selection process makes it possible to clarify unique exosome population, while undoubtedly leads to lower yields [37]. Since each method has its drawbacks, combinations of different methods are also used to improve the yields and purity of exosomes. For example, UC separates particles according to the size; a further density gradient fractionation step can separate components based on the density, thus removing co-isolated non-vesicular particles. Similarly, ultrafiltration and SEC are normally combined with UC or other methods to purify and enrich exosomes [38, 39].

### Exosome detection

Besides western blotting, enzyme-link immunosorbent (ELISA) is also widely used for detection of exosomal proteins. To date, multiple new techniques have been developed to detect exosomal proteins. For example, nanozyme-assisted immunosorbent assay (NAISA) captures exosomes by specific surface proteins and obtains signal by catalyzing a colorimetric reaction [40]. Besides colorimetric detection, fluorescence spectrophotometry is also widely used for substance identification and content determination. Wei et. al. recently developed exosome immunoassays using the Single Molecule array technology (SiMoa) and found that CD9-CD63 and Epcam-CD63 SiMoa assays specifically detect exosomes with high sensitivity and distinguish cancerous from non-cancerous plasma samples [41]. Recently, electrochemical biosensors have been developed as the changes in electrochemical signals can quantify elements such as antibodies which can recognize exosomes [42, 43]. Yoshioka et al. developed an analytical method to profile circulating exosomes directly from blood samples of colorectal cancer patients. Exosomes were captured by two types of antibodies and detected by photosensitizer beads, which enabled cancer-derived exosomes to be detected directly without a purification step. The system enabled detection of CD147 and CD9 double-positive EVs, which were abundantly secreted from colorectal cancer patients [44]. Recently, flow cytometry is emerging as a promising tool for the detection and sorting of exosomes [45]. Compared with conventional aldehyde/sulfate latex beads-based flow cytometry, the development of nano-flow cytometry, which enables single particle analysis, provides a new option to determine the heterogeneity of exosomes in body fluids [46].

Traditional ways of detecting and quantifying exosomal nucleic acid include quantitative real-time PCR (polymerase chain reaction), microarray, and RNA or DNA sequencing. However, exosomal nucleic acids are often of low abundance, largely affecting the sensitivity and accuracy of detection. To address these problems, droplet digital PCR, a newly invented technique, involves partitioning of PCR mixtures into abundant water-in-oil droplets and measuring each reaction independently [47]. Another new technique, fluorochrome-conjugated hairpin-like molecular beacon, can selectively hybridize with the exosomal target sequence and release the suppression on the fluorochrome, resulting in the emission of fluorescence signal. When combined with the nanopore detection technique, the exosomal nucleic acids can be detected without additional amplification. In addition, when combining molecular beacon with the imaging system based on total internal reflection fluorescence, the exosomal nucleic acid can be quantified on a single exosome level, and the results could provide insights on exosomal heterogeneity [48]. Besides, without the need of amplification or exosome isolation while using extremely small volume of samples, thermophoretic detection of nanoflare-modified sequence-specific aptamer can provide highly sensitive detection of exosomal nucleic acids [49]. For all the exosomal nucleic acid detection methods motioned above, multiple studies have shown that they are able to distinguish cancer patients form healthy subjects using plasma samples. Even though they are of high cost and requires prior knowledge of tumor-associated exosomal targets, molecular beacon-based detection system and thermophoresis-assisted detection platform could have huge applications in the clinic [50].

## Exosomes in cancers and their functions

**Fig. 2:**
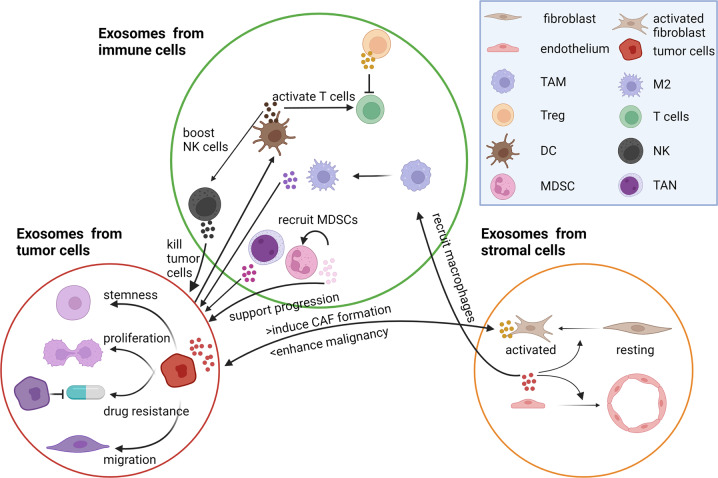
The interplay among stromal cells, immune cells, and tumor cells in the TME. Tumor cell-derived exosomes transfer malignant features to recipient cells, inducing the pro-tumoral roles of stromal cells and an immunosuppressive TME. Stromal cell-derived exosomes enhance the malignant properties of tumors cells in a feedback loop manner. Different types of immune cells play multifaceted roles through exosomes. TAM tumor-associated macrophage, Treg regulatory T cell, DC dendritic cell, NK natural killer cell, MDSC myeloid-derived suppressor cell, M2 M2 macrophage, TAN tumor-associated neutrophil, CAF cancer-associated fibroblast.

In the TME, the tumor cells, stromal cells and immune cells are closely related and consistently influence one another. The interplay among these cells through exosomes reshapes the tumor cells and reprogram the TME during cancer progression (Fig. 2).

### Exosomes derived from stromal cells

Stromal cells constitute the supportive tissue of the organs and consist mainly of fibroblasts and endothelial cells. Fibroblasts within the tumor are called cancer-associated fibroblasts (CAFs), the exosomes of which are proven to regulate tumor cell behavior, induce naive fibroblast differentiation and migration, and educate immune cells. For instance, compared to those from para-cancer fibroblasts, CAF-secreted exosomes may contain reduced miR-320a level, and this reduction may result in suppression of its downstream target PBX3 in the recipient cells, causing activation of ERK1/2 signaling and EMT phenotypic induction [2]. In addition, the intestinal fibroblast-derived exosomes have been reported to transfer epidermal growth factor (EGF) to support the survival of organoids and the formation of intestinal stem cell niche [3]. In addition to acting on tumor cells, the CAF-derived exosomes may also act on endothelial cells to facilitate blood vessel formation. For example, CAF-derived exosomes were found to contain vascular endothelial growth factor (VEGF), which binds to VEGFR2, activating the downstream signaling and promoting tube formation of HUVEC cells [51]. Exosomal miR-10a-5p from CAFs also facilitates angiogenesis and tumor cell growth [52]. Interestingly, the CAF-derived exosomes also signal feedback to adjacent naive fibroblasts to direct their differentiation [53]. For instance, platelet-derived growth factor (PDGF) can stimulate exosome production of hepatic stellate cells, and these exosomes enhance the migration of recipient hepatic stellate cells to exert their fibrogenic roles [54]. Under hypoxia or nutrient deficiency, mesenchymal stromal cells and CAFs may secrete exosomes that exert pro-tumorigenic roles through regulating tumor proliferation and apoptosis [6, 55]. Astrocyte-derived exosomes can lead to reversible PTEN loss of brain tumor cells, resulting in outgrowth of metastatic tumor cells via activation of NF-kB and production of CCL2 [56]. Compared with fibroblasts, the endothelial cell-derived exosomes are less studied. In line with the supportive role of their parental cells, endothelial cell-derived exosomes are shown to increase microvascular density, collagen deposition and macrophage infiltration [57] and promote the migration of hepatic stellate cells [58].

### Exosomes derived from immune cells

Immune cells in the TME are either resident in the primary tumor sites or recruited from bone marrow or circulation. Immune cells play crucial roles in recognition and elimination of tumor cells during cancer development; however, the tumor cells can direct their surrounding immune cells to be immunosuppressive to escape immune surveillance, causing disease progression. Therefore, immune cells have both anti-tumor and pro-tumor functions. Exosomes released from those immune cells may also present with multifaceted features. APCs, including dendritic cells (DCs) and macrophages, are critical mediators for antigen processing and presentation. DC-derived exosomes harbor MHC-bound antigens and co-stimulatory molecules, CD80/CD86 [59]. As such, DC-derived exosomes could partially or fully activate helper and cytolytic T cells to kill tumor cells [60, 61]. Besides, they could also induce monocyte differentiation to DCs and stimulate their maturation and release of functional exosomes [62, 63]. Moreover, DC-derived exosomes can also boost the activity of natural killer (NK) cells to induce apoptosis of tumor cells [9, 10]. Therefore, DCs-derived exosomes may suppress tumor progression, either directly by inducing T cell response or indirectly by recruiting and reprogramming other immune cells to combat against the tumor cells. Till now, DC-derived exosomes are applied in clinical trials for advanced NSCLC patients [64], and the therapeutic applications of exosomes will be discussed in a later section.

Tumor-associated macrophages (TAMs) are another important cellular component of the TME. TAMs include both anti-tumoral M1 and pro-tumoral M2 phenotypes. M2-derived exosomes are able to influence multiple functions of tumor cells, including drug resistance [65], proliferation [66] and metastasis [67]. Therefore, blocking M2-TAM-derived exosomes is a rational method for cancer therapy. In pancreatic ductal adenocarcinoma, it has been reported that hypoxia-induced an upregulation of miR-501a-3p in the M2-TAM-exosomes, and upon uptake of these exosomes, the tumor cells displayed accelerated proliferation, migration and invasion, and decreased apoptosis underlying activation of TGF-β signaling [66]. Besides tumor cells, M1 and M2 TAMs may also play contrasting roles on endothelial cells. M2-TAM exosomes could enhance angiogenesis to support tumor progression [66, 67]. Exosomes derived from THP-1 macrophages are able to suppress endothelial cell migration through internalization and degradation of integrinβ1 via Rab11^+^ endosomes [68]. In addition to TAMs, exosomes derived from tumor-associated neutrophil (TAN) are also biosynthesized and released on a “need” basis and their molecular composition is tailored to meet with the existing conditions. TAN-derived exosomes activate the oncogenic signaling of tumor cells through transfer of myeloperoxidase and neutrophil elastase [69].

Unlike the APCs, the functional killer cells, cytotoxic T cells and NK cells, directly attack tumor cells through contact-dependent cytotoxicity or cytokine release. Exosomes from these cells have been proven to achieve tumor elimination via similar modes of action [70]. In EBV-associated cancers, Vδ2-T-derived exosomes could significantly suppress tumor growth by inducing CD4^+^ and CD8^+^ T cell-mediated antitumor immunity [71].

There are also two types of immunosuppressive cells, namely myeloid-derived suppressor cells (MDSCs) and regulatory T cells (Tregs), and both are involved in tumor progression partially through exosomes. MDSC-derived exosomes can recruit MDSCs to the tumor sites through exosomal heterodimer S100A8/A9, which then activates NF-kB pathway. Similar to MDSC-derived exosomes that enhance immunosuppressive functions and enforce tumor development [72], the natural Treg cell-derived exosomes can inhibit CD8^+^ T cell responses and antitumor immunity [73].

### Exosomes derived from tumor cells (TEXs)

Exosomes from different cancer models recapitulate the organ specificity of their cell of origin. Tumor cells derived exosomes inherit the oncogenic or pro-tumoral properties and transfer them to the recipient cells. Exosomal fibronectin and tissue transglutaminase (tTG) are shown to be crucial to the oncogenic transformation of normal cells [74]. In addition, tumor cells with high metastatic potential could enhance the migratory ability of those with low metastatic potential, for example, through exosomal miR92a-3p [75]. Besides exchanging messages locally, the exosomes also transfer essential traits to distant organs.

TEXs not only transfer molecules to isogenic normal/tumor cells, but also act on immune cells to skew their polarization and maturation to form an immunosuppressive TME. TEX loaded with the N-terminus of HMGN1 (NIND) could activate DCs; these pulsed DCs significantly suppressed tumor growth through elevation of CD8^+^T cells and reduction of Treg cells in multiple cancer types, including hepatocellular carcinoma (HCC), pancreatic cancer and breast cancer [7]. Exosomes with higher expression of phosphatidylserine [76], migration inhibitory factor [4], and miRNA183-5p [8] are able to convert the surrounding phagocytes into TAMs and increase the inflammatory cytokine production. Furthermore, TEXs not only cause effects on the surrounding immune cells, but they also influence their recruitment and maturation.

Importantly, the tumor-stroma interaction via exosomes also involves reshaping of the TME. TEXs affect the differentiation and proliferation of stromal cells in both primary and secondary sites. For instance, exosomes from the tumor cells under hypoxia can upregulate caveolin-1 (CAV1), which is responsible for CAF-like differentiation [77]. Also, exosomes packaged with TGF-β could transform normal fibroblasts to CAFs through upregulating fibronectin expression [78] or through Erk1/2 and Akt signaling [79]. Besides, the tumor cells with stemness properties and high metastatic potential display stronger effects on the adjacent and distant fibroblasts. It has been reported that CD44^+^ colorectal cancer cells were able to secrete more exosomes than CD44^-^ cells without affecting the miRNA contents. These TEXs from cancer stem cells could activate fibroblasts and support their proliferation. Therefore, the number of exosomes might be important in fibroblast activation in the context of CRC [80]. Functionally, these CAFs polarized by TEX could further alter the tumor cells towards more malignant properties in a feedback loop manner. TEXs also contribute to angiogenesis through recruiting and activating endothelial cells [81]. In HCC, metastatic cell-derived exosomes contained higher levels of Nidogen 1 and complement factor H and promoted tumor progression through developing more aggressive tumor behavior and angiogenesis as well as lung metastasis through increased pulmonary endothelial permeability and activated fibroblasts [82, 83]. Taken together, these findings underscore the stroma-tumor interaction through exosomes in tumor progression.

## Potential use of exosomes as liquid biopsy in cancers

Traditionally, tissue specimens are routinely used to provide clinical diagnosis, prognosis and assessment for molecular changes. However, the tumor tissues obtained are subject to sampling bias because of intratumoral heterogeneity. Tissue samples may also be difficult to obtain due to many reasons. In comparison, liquid biopsy has emerged and may overcome the shortcomings of traditional tissue biopsy [84–86]. Liquid biopsies are non-invasive, can be obtained serially, and may facilitate early cancer detection. Meanwhile, exosomes are secreted by all types of cells and exist in biological fluids particularly in blood, rendering them as potential biomarkers to diagnose cancer and monitor response to treatment. Cancer-derived exosomes as biomarkers for early cancer detection is promising since exosomes could reveal genetic or phenotypic alterations in cancer cells of origin [11, 12, 87].

### Exosomal proteins as potential biomarkers for cancers

Secretion of exosomes has been reported to be increased in patients with cancer; hence exosomal markers are emerging as attractive targets for cancer detection [88–90]. To this end, studies have analyzed the relationship between the amount of plasma exosomes and tumor burden [91–94]. Study on stage IV oral SCC patients showed that the levels of CD63-positive and caveolin 1 (CAV1)-positive exosomes were upregulated in patients and associated with poorer prognosis [95]. In prostate cancer, higher levels of exosomal CD81 and prostate-specific antigen were observed, which could distinguish prostate cancer patients from benign prostatic hyperplasia and healthy subjects [96]. Jakobsen et al. developed an extracellular array to assess the level of 37 lung cancer-related proteins in exosomes. The assay was reported to have 75% accuracy in distinguishing NSCLC patients from healthy individuals, using only 10 μL plasma sample [97].

Tumor-derived exosome proteins have been reported to be important functionally and prognostically in cancer development and immune responses [4, 98, 99]. Mass spectrometry analyses are mostly applied for the identification of different proteins in blood exosomes derived from cancer patients and healthy subjects. Investigation on surface proteins of exosomes from pancreatic ductal adenocarcinoma revealed multiple specific biomarker candidates (CLDN4, EPCAM, CD151, LGALS3BP, HIST2H2BE, and HIST2H2BF) [100]. Glypican 1 (GPC1) is one of the most investigated surface markers of exosomes derived from pancreatic and colon cancer [11, 101]. The level of GPC1^+^ circulating exosomes was shown to distinguish healthy subjects and patients with benign pancreatic disease from patients with early- and late-stage pancreatic cancer. The levels of GPC1^+^ exosomes are associated with the tumor burden and patient survival after surgery, thus enabling the detection of pancreatic cancer and possibly response to therapy [11]. There are studies suggesting GPC1^+^ exosomes in combination with serum CA19-9 could serve as a diagnostic marker for pancreatic cancer [102, 103].

Evaluation of phosphoproteins in exosomes was of interest, as protein phosphorylation is a common cellular regulatory machinery in controlling protein functions [104]. Chen et al. identified more than 100 phosphoproteins in plasma exosomes that are significantly higher in patients with breast cancer compared to healthy individuals [105]. This study indicates that the detection of phosphoproteins in exosomes may facilitate cancer screening and monitoring.

Recently, a purification system for EVs was developed based on multi-marker antibody cocktails for HCC, which may pave the way for its early detection. In the study, trans-cyclooctene-conjugated EpCAM, ASGPR1 and CD147 antibodies were applied in the cocktails for the purification of HCC exosomes [106]. However, one limitation to this approach is that preparation of such multi-marker antibody cocktails relies on solid understanding on cancer-specific exosome markers. Emerging studies have focused on pan-cancer circulating protein markers such as EpCAM and carcinoembryonic antigen (CEA); both proteins were found to be highly enriched in exosomes derived from colorectal adenomas and cancers [15, 107]. Besides, CD24 and EGFR have also been characterized in exosomes derived from ovarian cancer patients and proposed to be potential biomarkers for ovarian cancer [108]. In prostate cancer, prostate membrane-specific antigen, which has been used to detect prostate-specific exosomes, was reported to be increased in prostate cancer patients [109, 110].

To better classify the tissue and plasma derived EVs including exosomes, Hoshin et al. compared the proteomic profile of EVs derived from tumor tissue and plasma to identify cancer-specific protein signatures of EVs. As a result, they found that proteins in the EVs, such as VCAN, TNC, and THBS2, could distinguish tumors from normal tissues. Furthermore, they defined a panel of tumor-type-specific proteins in the EVs derived from tumor tissue and plasma, which could help classify tumors of unknown primary origin. The study indicates that EV proteins may serve as reliable biomarkers for cancer detection and diagnosing the cancer type [111].

Besides early diagnosis and prognosis, exosomal proteins are attractive candidates for personalized treatment and post-treatment disease monitoring. Recently, emerging evidence has revealed that exosomal PD-L1 contributes to immunosuppression, which is a potential predictor for anti-PD-1 therapy in melanoma and NSCLC [19, 20, 112–114]. In contrast to the heterogeneous PD-L1 expression in tumor tissues and the invasive nature of tumor biopsy, exosomal PD-L1 as a blood-based biomarker is an attractive option.

Although the potential of exosomal proteins as cancer biomarkers is promising, the identification and quantification of exosomes in clinical samples remain challenging. As mentioned before, Yoshioka et al. developed an analytical method, which profiles circulating exosomes directly from blood samples of colorectal cancer patients without a purification step. Such new liquid biopsy technique that sensitively detects cancer-specific exosomes will facilitate the diagnosis of cancer [44].

### Exosomal miRNA as potential biomarkers for cancer

As dysregulated miRNA levels have been described in many human malignancies, and as tumor-derived exosomes reflect the miRNA expression of originating tumor cells, different miRNAs from tumor-related exosomes have been evaluated as biomarkers in the plasma of tumor patients, aiming to improve cancer diagnosis and treatment.

Elevated miR-21 in circulating exosomes has been reported to be a potential biomarker in several malignancies such as liver, gastric, breast, colorectal, ovarian and esophageal cancer, and increased level of exosomal miR-21 derived from urine has been associated with bladder and prostate cancer [115–120]. Here, we take liver cancer as an example. Exosomal miR-21 may distinguish HCC patients from chronic hepatitis B or liver cirrhosis patients, although there have been contradictory results [121, 122]. Furthermore, many other types of miRNA have been reported to be found in serum-derived exosomes from HCC patients and some were reported to be positively associated with HCC progression and poor survival rates [122, 123]. On the other hand, some miRNAs such as miR-638 were shown to be downregulated in HCC patients and negatively associated with more aggressive tumor behavior with advanced TNM stage, tumor size and vascular invasion [124–128]. The combination of multiple microRNAs may enhance the diagnostic and prognostic potential of exosomal miRNAs.

In addition to early diagnosis and prognosis, exosomal miRNAs have also been evaluated in their predictive ability to patients’ therapeutic response and outcome in a wide variety of malignances. It has been reported that elevated level of exosomal miR-146a-5p was a potent predictor for response to cisplatin, while increased levels of exosomal miR-425-3p and miR-96 were predictive of cisplatin resistance in lung cancer [129–131]. In addition, high miR-155 and miR-301 levels in circulating exosomes were correlated with breast cancer patients’ complete response upon neoadjuvant therapy [132]. In serum exosomes, elevation in levels of miR-155, along with miR-301 and miR-339-5p, was reported to predict gemcitabine resistance in pancreatic ductal adenocarcinoma, responsiveness to neoadjuvant therapy in breast cancer and pre-operative radiotherapy in locally advanced esophageal SCC, respectively [132–134]. In addition, serum exosomal miR-718 was negatively correlated with the recurrence rate for HCC patients [127]. Using exosomal miRNA levels as modern molecular diagnostics is appealing for individualized personalized treatment and post-treatment disease monitoring.

Although the utility of circulating exosomal miRNAs as cancer diagnostic and prognostic markers is continuously evolving, applying exosomal miRNA signatures in blood needs to be prudent. Though highly valuable, the detection of exosomal RNAs in early-stage cancers remains challenging since those RNAs are of low expression levels. To this end, a method using nanoparticle-based biochips that can capture circulating EVs without isolation has been reported. It lights up encapsulated RNAs and amplifies signals in situ in one step, and this should be advantageous for early cancer detection [135].

### Other exosomal components as potential biomarkers for cancers

Emerging evidence has revealed that abnormal expression of long non-coding RNA (lncRNA) contributes to tumorigenesis and metastasis, and lncRNAs are selectively sorted into cancer-exosomes [136]. Since the expression of many lncRNAs is tissue-specific [137], profiling exosomal lncRNAs may facilitate cancer diagnosis. For instance, lncRNA-ATB was identified as a prognostic marker in combination with miR-21 in HCC [138]. Similarly, serum exosomal lncRNA-UCA1 and HOTTIP are potential diagnostic biomarkers for bladder cancer and gastric cancer, respectively [139, 140].

Circular RNA (circRNA) is a novel member of noncoding RNA that has been identified in exosomes [141]. Li et al found that serum exosomal circRNA was able to distinguish patients with colon cancer from healthy controls [142]. Double-stranded DNA (dsDNA) was also characterized in tumor-derived exosomes; interestingly, the exosomal dsDNA was found to represent the entire genome and reflect the mutational status of the parental tumor cells [143, 144].

There is increasing evidence supporting that, in contrast to nontumorigenic cells, many tumor cells expose phosphatidylserine on their surface [145–147]. To that end, Sharma et al. developed an ELISA-based system that detects picogram amounts of exosomal phospholipid in the plasma as a cancer biomarker, which was capable of discriminating breast cancer-bearing mice from normal control [148]. A similar study found that patients with ovarian cancer exhibited much higher levels of PS-positive exosomes compared to the healthy controls [149].

For cancer patient management, the identification of novel noninvasive biomarkers to inform early diagnosis and disease prognosis, support personalized treatment selection, and monitor therapeutic progression is of high priority. The possibility of combining protein, RNA, and lipid in exosomes could potentially increase the precision and sensitivity of exosome for cancer diagnosis and prognosis.

## Exosome-related cancer therapy

Being double-layered, nano-sized, cell-free, and having their host-derived nature, exosomes are able to transport cargoes to designated target cells with highly specific biodistribution and low immunogenicity [150]. As a result, exosome-associated treatments are potentially promising against cancer. In general, three exosome-related approaches were utilized: 1. depletion of tumor-derived exosomes using exosome inhibitors, 2. administration of specific cell types-derived exosomes, and 3. engineering exosomes as a vehicle to carry antineoplastic agents to selective target sites.

### Depletion of tumor-derived exosomes using exosome inhibitors

As mentioned in previous sections, cancer cell-derived exosomes were found to facilitate the various stages of cancer metastasis and promote cancer drug resistance. Therefore, depletion of cancer cell-derived exosomes would have therapeutic benefits in cancer patients. Current established exosome inhibitors are designed to target exosome biosynthesis or trafficking pathways. Examples of drugs inhibiting exosome biogenesis include cholesterol synthesis inhibitor D-pantethine, neutral sphingomyelinase inhibitor GW4869, and Rab27A inhibitor tipifarnib. Inhibitors of exosome trafficking include Ras inhibitor manumycin A, cytoskeleton reorganizing ROCK inhibitor Y27632, and cysteine proteinase inhibitor calpeptin. Studies have been done on the anti-cancer effects of some of the exosome inhibitors in vitro and in vivo [151, 152]. As an example, cancer-derived exosomes were found to express a high level of PD-L1, resulting in exhaustion of T cell activity and the subsequent tumor resistance against immune checkpoint inhibitors. Using mouse models, Wang et al. showed that elimination of cancer-derived exosomes using GW4869, along with ferroptosis inducer, significantly inhibited exosomal PD-L1-mediated immunosuppression, restored anti-tumor immune response, and reduced metastasis [153]. Other compounds of exosome inhibitors, such as Rab27A inhibitors, have also been tested [151, 154]. However, the aforementioned direct exosome inhibitors were found to be too toxic to be used clinically. Proton pump inhibitors (PPIs) can indirectly inhibit exosome secretion. They were shown to impair exosome release and, at the same time, promote chemotherapeutic drug retention within the tumor cells [155]. Besides PPIs, extracorporeal hemofiltration could also be used to eliminate circulating tumor derived exosomes. Clinical trials have been conducted on less-selective Prosorba Column, which is a plasma filtering device capturing immune complexes containing IgG, but only few patients were shown to have clinical benefits. More advanced filtration system with higher target specific affinity, such as adaptive dialysis-like affinity platform technology, are under development and seem suitable against cancer metastasis. However, regimen optimization and efficacy testing are required before application in the clinical setting [155].

### Administration of specific cell types derived exosomes

Exosomes derived from non-tumor cells may be used as therapeutic agents against tumors. DCs, mesenchymal stem cells, and many other cells were used to produce the exosomes, each with unique anti-tumor functions. DC-derived exosomes not only express tumor antigen-presenting MHC molecules, but they also are resistant toward tumor-mediated immunosuppression [156]. Two approaches are developed to further enhance the stimulating function of DC-derived exosomes. DC could be stimulated with TNF-α, granulocyte-macrophage colony-stimulating factor or IL-4, and the exosomes released would express various immunostimulatory surface proteins, such as MHC and CD86 to stimulate T cells [157]. DC could also be treated with DC-specific surface proteins antibodies coupled with tumor antigens, and the treated DC-derived exosomes have been shown to have significantly boosted antigen presentation efficiency [156]. The tumor-specific antigen presented on DC-derived exosomes can directly bind to the T cell receptors or be taken up by recipient cells, which later present the antigens on their membranes, with or without processing [158]. Many Phase I and II clinical trials have been conducted, testing the safety and efficacy of either stimulated DC-derived exosomes or those pulsed with tumor-associated antigens. Exosome administrations were well tolerated, with little or no grade 2 and only 1 case of grade 3 hepatotoxicity adverse events reported. Interestingly, activation of NK cell functions was observed after exosome administration in multiple trials. In most trials, patients’ immune responses were shown to be boosted after the exosome treatment. Nevertheless, to avoid T cell tolerance and enhance clinical response rate, improved exosome targeting specificity or combining exosome treatments with immune checkpoint blocking drugs are required [150, 156, 159, 160].

Besides DCs, mesenchymal stem cells, due to their immune-modulating effects and high efficiency in exosome production, are also popular choices [161]. Clinical trials have also been conducted on mesenchymal stem cell-derived exosomes, and they were shown to be safe and well tolerated [160, 162]. Exosomes can also be derived from ascitic fluid. In one colorectal cancer clinical trial, exosome was used in combination with granulocyte-macrophage colony-stimulating factor as adjunct therapy, were found to elevate cytotoxic T lymphocyte level and function in patients. The effect, however, was diminished for those who received exosomes solely [163]. As another example, even though most research agreed on the immunosuppressive and metastasis-promoting effect of exosomes derived from TAM, it was found that exosomes isolated from TAMs of mouse adenocarcinoma tumor promoted the proliferation, activation and functions of both CD4^+^ and CD8^+^ T cells in vivo [164]. Whether TAM-derived exosomes could boost anti-tumoral T cells in the TME remains to be further investigated. More thorough examination and clinical trials are needed for those exosomes.

### Exosomes as potential vehicles for drugs

Recently, parental exosomes or artificially modified exosomes used as a drug delivery vehicle have become appealing to researchers. Chemotherapeutic drugs, or tumor-targeting RNAs or proteins, are packed inside exosomes in order to achieve more effective biodistribution. Exosomes are mostly synthesized from dendritic cells, mesenchymal stem cells, macrophages, bovine milk, or even cancer cells, as exosomes released by those cells can either present tumor-associated antigens, modulate the immune response, high-yielding, or be biologically more stable or penetrating [165]. Then chemotherapy drugs or RNAs are encapsulated into exosomes by mixing, followed by either electroporation or sonication [165–167]. Other membrane engineering methods, such as hybridizing concentrated exosomes with modified liposomes were also developed. Using this method, the hybridized exosomes still maintained their exosomal features, obtained a high drug loading and releasing efficiency, and at the same time had a higher yield compared to exosome isolation technique alone [22]. Many in vitro, in vivo, and even clinical studies have been done on MSC-derived and DC-derived exosomes, while loaded with cancer-targeting anti-miRNA/miRNAs; those exosomes were found to be effective in inhibiting cancer growth, metastasis, and drug resistance as well as induce NK and T cell responses [160, 161, 168].

One of the proposed crucial benefits of using exosomes as anti-cancer factor vehicles is target cell specificity. Tumor cells were found to internalize more exosomes than normal cells, and internalization of exosome is more efficient than that of similar-sized liposomes; without additional modification, exosomes have a high selectivity to start with [161]. In addition, parental cells could be modified so that the secreted exosomes could express specific surface proteins and achieve an even higher target selectivity. As an example, Bellavia et al. [169] modified HEK293T cells to secret exosomes that expressed proteins with the IL3R ligand domain, which bound IL3R on chronic myeloid leukemia (CML) cell membrane. Since CML drug resistance is usually acquired by overexpression of or mutation in tyrosine kinase BCR-ABL, 293T-derived IL3R-expressing exosomes were encapsulated with BCR-ABL inhibitor imatinib or BCR-ABL siRNAs, and they were shown to inhibit CML growth and drug-resistance in vivo. Similarly, to reverse the potential resistance against 5-fluorouracil in colorectal cancer therapy, Liang et al. [170] electroporated both 5-flurouracil and miR-21 inhibitor into modified 293T-derived Her2-LAMP2-expressing exosomes. Both miR and 5-flurouracil-containing exosomes were shown to bio-distribute mostly to the target tumor, suppress tumor growth, and have no significant renal or hepatic toxicity in vivo. Recently, more studies have been done, attempting different approaches that could successfully enhance exosome selectivity, by expressing target-specific ligands, anchoring superparamagnet nanoparticles, and more [150]. Several Phase II clinical trials on antineoplastic agents loaded with exosomes were also conducted to treat metastatic pancreatic cancer or malignant ascites and pleural effusion. The outcomes of those trials, however, are not yet available [160].

In general, exosome-related therapy could be beneficial in cancer therapies, thanks to their low immunogenicity, safety, preferred tumor homing, and effective recipient cell targeting. However, utilizing exosome-related techniques in the clinics remains challenging. It is difficult to standardize exosome separation and production, since no current isolation method can achieve high-yield, high recovery and specificity, time-saving and low-cost all at the same time, and likewise, different cell-derived exosomes have their unique features and funtions [160, 165, 171]. Furthermore, even after modifying the exosomal protein coating to prolong exosome’s half-lives (such as by PEGylation, CD55 and CD59 expression, and albumin coating), the clearance rates for exosomes are still high [165, 172], and this posts bigger demand for high exosome yields. Other challenges also exist, such as the difficulties to develop exosomes from patients, the heterogeneity of disease malignancies, complexity of TME, and mixed effects of exosomes.

## Discussion

Currently, exosome biology is studied in cell culture systems and mouse models; however, there is a need for experiments to mimic physiologically relevant conditions. Exosomes isolated from cell culture systems are usually obtained from large numbers of cells outside the physiological range [120]. Relevant animal models which allow study on biogenesis, trafficking, and cellular entry of exosomes are very much needed. As mediators of intercellular communication, exosomes are emerging as potential tools for both diagnostic and therapeutic purposes. Numerous studies have identified multiple exosomal miRNAs and proteins which have the potential to be biomarkers for cancer diagnosis, prognosis, and prediction of therapy responses. However, currently, due to the different methods of exosome isolation, different study data cannot be compared, making it difficult to evaluate their clinical applicability [119]. Thus, there needs to be standardization of the methods and procedures in the isolation and purification, which should be simple and reproducible. Moreover, since tumor cell-derived exosomes only represent a small fraction of all exosomes in body fluids, detection of exosomes with high sensitivity are required for exosome-based cancer diagnostics. Although various methods have been developed for exosome isolation and detection [50, 173], which are superior to conventional methods, new platforms still face the challenges of limited yield, low specificity and sensitivity, and high heterogeneity of different exosome subsets. Single exosome detection and identification should improve our understanding of exosomes derived from different origins, including unique molecular profile of cancerous exosomes. All these techniques would promote the development and reproducibility of exosome-related cancer diagnosis and therapies. On the other hand, improvement in the technology for one-step exosome detection without isolation would very much advance the biomarker discovery of exosomes [44, 135].

For exosomes loaded with therapeutic molecules such as specific miRNA, siRNA and recombinant proteins, as well as synthetic small-molecule drugs, they may confer optimal bioavailability and minimal immune rejection. However, the complicated composition and functional activity of natural exosomes may limit their clinical applications. Standardization in exosome loading is also urgently needed. A promising alternative is to develop artificial nanoparticle or liposome which could mimic the features of natural exosomes. Although facing similar challenges of natural exosomes, the development of artificial exosomes will have commercial advantages such as higher yield, easier quality control, more reproducibility and larger-scale productivity. Importantly, the critical components of exosomes for therapeutic delivery need to be clarified, and the strategies for obtaining artificial exosomes need to be standardized. Currently, natural exosomes are studied in just preliminary clinical trials, while artificial exosomes have not yet been translated into clinical application [174]. The development of exosome-related drug delivery needs contribution from multidisciplinary effort.

In summary, exosomes derived from tumor, stromal and immune cells contribute to the multiple stages of cancer progression as well as resistance to therapy. Since specific contents within exosomes originate from their cells of origin, this property enables exosomes to function as valuable diagnostic and prognostic biomarkers. Furthermore, the applications of exosomes as direct therapeutic targets or engineered vehicles for drugs may open up new avenue for therapy. Better understanding of exosome biology will certainly help pave the way to exosome-based clinical applications.

## Data Availability

Data sharing not applicable to this article as no datasets were generated or analysed during the current study.
